# Modeling of Respiratory Motion to Support the Minimally Invasive Destruction of Liver Tumors

**DOI:** 10.3390/s22207740

**Published:** 2022-10-12

**Authors:** Dominik Spinczyk, Sylwester Fabian, Krzysztof Król

**Affiliations:** Faculty of Biomedical Engineering, Silesian University of Technology, 40 Roosevelta, 41-800 Zabrze, Poland

**Keywords:** destruction of liver tumors, modeling of respiratory motion, image-guided navigation, minimally invasive liver interventions

## Abstract

Objective: Respiratory movements are a significant factor that may hinder the use of image navigation systems during minimally invasive procedures used to destroy focal lesions in the liver. This article aims to present a method of estimating the displacement of the target point due to respiratory movements during the procedure, working in real time. Method: The real-time method using skin markers and non-rigid registration algorithms has been implemented and tested for various classes of transformation. The method was validated using clinical data from 21 patients diagnosed with liver tumors. For each patient, each marker was treated as a target and the remaining markers as target position predictors, resulting in 162 configurations and 1095 respiratory cycles analyzed. In addition, the possibility of estimating the respiratory phase signal directly from intraoperative US images and the possibility of synchronization with the 4D CT respiratory sequence are also presented, based on ten patients. Results: The median value of the target registration error (TRE) was 3.47 for the non-rigid registration method using the combination of rigid transformation and elastic body spline curves, and an adaptation of the assessing quality using image registration circuits (AQUIRC) method. The average maximum distance was 3.4 (minimum: 1.6, maximum 6.8) mm. Conclusions: The proposed method obtained promising real-time TRE values. It also allowed for the estimation of the TRE at a given geometric margin level to determine the estimated target position. Directions for further quantitative research and the practical possibility of combining both methods are also presented.

## 1. Introduction

Liver tumors are a serious health concern, and are classed as benign or malignant [1]. Primary liver cancer is globally the sixth most frequent cancer and the fourth leading cause of death from cancer [2]. In 2018, it occurred in 841,000 people and resulted in 782,000 deaths globally [2].

Image navigation systems allow for more precise treatments of liver tumors using minimally invasive techniques, reducing the invasiveness and duration of therapy and increasing its usefulness [3]. The use of these systems in clinical practice can be divided into stages. The first step in using an image navigation system is to prepare the patient’s anatomical model based on pre-operative images. Typically, three-dimensional computed tomography (CT), magnetic resonance (MR), or ultrasound images (US) are used. Contrast is usually used to accurately differentiate focal lesions. For radiotherapeutic protocols, a 4D CT or MRI series is used, which contains additional information on organ mobility and focal changes during the respiratory cycle [4,5]. Through the use of image segmentation algorithms, a personalized anatomical model of the patient is created, containing labeled and subdivided anatomical structures and pathological features relevant to the potential safety and effectiveness of surgery. Once a personalized model of the patient’s anatomy has been created, the surgeon can progress to the second stage of planning the procedure, including selecting which therapeutic techniques are most appropriate. The most important and most difficult stage from the point of view of the procedure’s effectiveness is the operative support stage, during which the pre-operative anatomical model of the patient is correlated with the patient’s position during the procedure [3,6]. At this stage, there may be local organ deformities that the static pre-operative model has not captured but are necessary to take into account during the procedure.

In the case of the liver, where the performance of minimally invasive procedures is a challenge due to the organ’s size, the main cause of local deformities is the movement of the organ during respiration due to its adherence to the diaphragm. However, there are various ways to overcome the problem of respiratory movements during procedures:

- Breath-holding—this requires the patient to hold their breath during inhalation or expiration. This requires close cooperation between the patient and the doctor, which is not always possible due to the patient’s condition and accompanying stress.
- The tracking of respiratory movements—in this case, the patient can breathe freely.
- Gating—the patient can breathe freely, and procedural activities are performed in a specific phase of the respiratory cycle, selected when planning the procedure. According to reports from the American Society of Medical Physicists, this method is used by many cancer hospitals [7].

Information about the phase of the respiratory cycle alone is not sufficient. There is also a need to consider internal organ movements [8]. An estimate of soft tissue deformation can be made using non-rigid recording algorithms. One of the most frequently used classification criteria for non-rigid recording algorithms is the form of transform representation. Using this criterion, we can distinguish parametric and non-parametric algorithms of non-rigid registration.

Parametric transformations are represented as deformation fields defined by a linear combination of classes of base functions. Various forms of base functions are used: B-splines, radial basis functions, thin plate splines (TPS), Wendland functions, and irregular grids.

Non-parametric transformations depend on the physical properties of the medium and the form of the function chosen for the registration process. Known classes of non-parametric transformations include linear elastic, viscoelastic, hyperelastic, Navier–Stokes equation, and Demons algorithm.

In the literature, there have been attempts to estimate liver displacement based on MR spatial sequences [9]. Rijkhorst et al. proposed estimating the position of the liver volume voxels in any phase of the respiratory cycle by interpolating the spline curve of the positions of the displacements obtained in the recorded liver positions. He obtained an improvement in the mean liver localization error ranging from 4.6 ± 3.8 mm to 2.5 ± 1.7 mm after correction. However, it is worth paying attention to the time constraints of such calculations. Adjusting the time sequence for one patient took approximately 30 min, using the differential equation system module of the ANSYS package [10].

Soft tissue deformations are simulated using a wide range of mathematical methods—from the spring pendulum model to the so-called free-form deformation (FFD) and finite element method (FEM), to combinations of the FEM with methods based on a linear or non-linear theory of elasticity. An example of a mathematical model of the liver based on the FEM method is an algorithm that uses a biomechanical model to describe organ displacement during surgery [11]. The average accuracy of the model is approximately 2 mm, and the computation time is 5 to 7 min. Preparation of the model requires manual marking of the organ boundaries.

To sum up, difficulties in the numerical modeling of liver tissue deformation include the computational complexity of algorithms, the lack of direct determination of the mechanical parameters of the tissue, large fluctuations in the coefficients found in the literature, a lack of direct correlation between studies carried out on animal and human organisms, a large variability of the range of motion depending on location within the organ, individual anatomical features of the patient, a variability of these features between subsequent treatments, and the difficulty of verifying the results obtained for a specific patient. Considering the above limitations, this article aims to present a method of estimating the displacement of the target point using non-rigid recording algorithms working in real time.

The novelty of the proposed work is the presentation of the method of combining two methods and unique results, which allows to estimate the location of the target point by modeling the deformation field and combining imaging information from the preoperative model and from intraoperative ultrasound images. Ultimately, this reduces the challenge of visibility of a pathological lesion during a minimally invasive percutaneous abdominal intervention. At the same time, the proposed approach takes into account the most important factor hindering the identification of the pathological lesions, which is also the main source of deformation of organs during the procedure, which are movements caused by the patient’s respiratory process.

## 2. Materials and Methods

### 2.1. Modeling of Respiratory Movements as a Problem of Time–Space Adjustment

Modeling of the target point location and displacement is carried out using the proposed method:

- Initial global registration of coordinate systems;
- Measurement of the position of markers on the patient’s body;
- Determination of the time course of the marker registration error;
- Determination of the deformation field;
- Optimization of the selection of parameters for determining the deformation field;
- Estimation of the patient’s respiratory phase;
- Adaptation of the AQUIRC approach.

**Figure 1:**
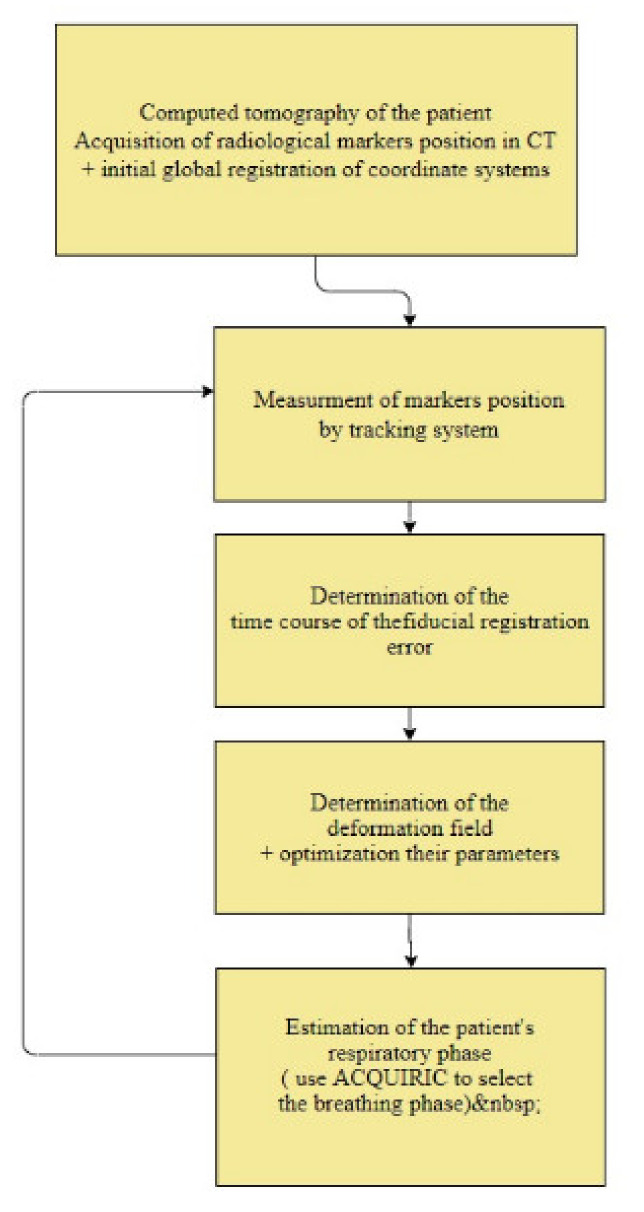
The flow diagram of the method of estimating the displacement of the target.

Figure 1 presents the flow diagram. The individual steps will be detailed below.

#### 2.1.1. Initial Global Registration of Coordinate Systems

To determine the real-time position of the patient’s body during the procedure, specially designed markers are used, which are attached to the skin surface when preparing the patient for diagnostic testing or surgery. During the procedure, their position is detected by a position tracking system.

The markers are specially designed and clearly detectable in CT images and by video-metric position tracking systems, for example, ClaroNav Claron Hx 40 [12].

#### 2.1.2. Measuring the Position of Markers on the Patient’s Body

The next step in minimizing fit errors is to pre-register the global set of marker positions on the patient’s body to the marker set in the pre-operative anatomical model. The research analyzed the results obtained through the use of two mechanisms, rigid registration, and Horn’s algorithm [13], where the transformation takes the form:

(1) $$X^{\prime }=R\;\cdot \;\left(X-C\right)+V+C$$

where: R—rotation matrix, C—rotation center matrix, and  V—offset translation matrix. All matrices are 4 × 4. The translation matrix T is given by the formula:

(2) T=V+C−R · C

Affine registration [8,9], which, apart from translation and rotation, uses scaling and curvature operations and is expressed by the formula:

(3) $$X^{\prime }=R\;\cdot \;\left(S\;\;\cdot \;Sh\;\cdot \;X-C\right)+V+C$$

where: S—scaling matrix and Sh—slope matrix. Both matrices are 4 × 4.

#### 2.1.3. Determination of the Time Course of the Marker Registration Error

1. The position of the markers on the patient’s surface during the respiratory cycle is measured.

Determination of time courses in the respiratory cycle of the Fiducial Registration Error (FRE; the error of the fiducial markers following registration;

$$∥T\left(p_{f}\right)-p_{m}∥$$

where *T* is the estimated transformation and the points pf and pm were used to estimate *T*.

#### 2.1.4. Determination of the Deformation Field

The assumption of no shape change is a simplifying assumption; therefore, local transformations, or rather combinations of these transforms, are also used for recording based on a set of points. Then, the transformation equation is presented in the form [14,15]:

(4) $$F=C^{T}\cdot G\left(x\right)+Rot\cdot x+Trans$$

where: $C^{T}$—vector of weights, G—base functions for each point, *Rot*—rotation matrix (also including the scaling factor), *Trans*—translation vector, and *F(x)*—set of independent deformation field functions for a point at x position

(5) $$F\left(x\right)={\left[\;f_{1}\left(x\right),\;\ldots ,\;f_{\dim}\left(x\right)\right]}^{T}$$

The most commonly used base functions are thin plate splines (TPS) and elastic body splines (EBS).

##### TPS Spline Curves

The TPS algorithm, the basics of which were presented by Harder and Desmarais [11], can be described as an interpolation equation for a given number of N markers in the position tracking system, $\vec{p_{i}},\;$ and in the CT images, $\vec{q_{i}}$, where i=1,2,…,N, about d-dimensions.

##### EBS Spline Curves—Interpolation Approach

Similar to TPS, the EBS approach is a mechanism that allows landmarks to be mapped from one coordinate system to another, taking deformation into account [16]. Unlike TPS, where the observations of thin sheet metal deformations were used to create the mathematical foundations, the EBS algorithm models deformations of a homogeneous, isotropic, and elastic body (e.g., rubber) under the influence of a force field. It is based on the partial differential equation (PDE) presented by Navier [12,13], which describes the deformation of objects built using materials with the properties described above.

#### 2.1.5. Optimization of the Selection of Parameters for Determining the Deformation Field

In the approximation approach, the EBS algorithm described in the previous sections takes two parameters into account: the α coefficient (calculated using Poisson’s ratio ν) and λ. Their selection determines the quality of mapping displacement of organs with respiratory movements [17,18].

In order to select the optimal values of these parameters, the set of marker positions was divided into two subsets with a similar number of samples—training and testing.

In the training subset, one marker and its counterpart from the CT coordinate system were selected as the target marker for each time moment in which the positions of markers were recorded in the position tracking system. Then, the selection of parameters α and λ was optimized using the particle swarm optimization (PSO) algorithm, so that the TRE measured with the target marker was as small as possible. The calculations were performed on each registered sample, and the moments when the FRE was minimal within one respiratory cycle were determined. Finally, the values of the parameters α and λ calculated for these moments were averaged and adopted as constants for the EBS calculations on the test set.

##### PSO Algorithm

PSO was presented in 1995 by Kennedy and Eberhart [19]. Its assumptions are based on the observation of flocks of flying birds looking for food or shoals of fish, presented by Reynolds [20]. Heppner and Grenander [21] simulated this type of movement and described the mechanisms that allow the birds to change the direction of flight synchronously. The mathematical model assumes that birds avoid collisions and try to maintain an optimal distance from their neighbors during the flight. Moreover, the PSO considers Wilson’s hypothesis [22] based on research on shoals of fish, whereby individuals in a population can benefit from the discoveries and experiences of others.

##### Algorithm of Swarm Optimization—Differential Evolution

This algorithm was selected based on a thorough literature review. In the literature, tests comparing the effectiveness of swarm optimization algorithms and the differential evolution algorithm obtain the best results [23].

Differential evolution is a simple yet effective evolutionary algorithm proposed by Storn and Price [24,25].

The scheme for differential evolution is described below as a pseudocode. It processes the population $X=\left\{\vec{x_{1}},\vec{x_{2}},\;\ldots ,\;\vec{x_{N}}\right\}$ composed of N vectors or individuals. According to the typical scheme of evolutionary algorithms, after a random initiation of a population, transformation occurs due to genetic changes such as mutation and crossing. It then undergoes a selection mechanism. Storn et al. proposed several variants of the differential evolution algorithm, introducing at the same time notation DE/𝑋/𝑌/𝑍. Storn et al. proposed several variants of the differential evolution algorithm [26].

#### 2.1.6. Estimation of the Patient’s Respiratory Phase

For data analysis, a patient record structure was designed to store the required information. To register the position of the markers from the position tracking system to the CT coordinate system, the position of the corresponding points in both coordinate systems must be correlated. To determine the position of the markers in the coordinate system associated with the CT images, an application was designed that allows the radiologist to view the exam, navigate individual sections in any view, locate radiographic markers, and record their positions in a configuration file.

Depending on the patient’s anatomy (e.g., abdominal size), previous procedures, and presence of postoperative scarring, seven to ten correlated radiographic markers were positioned on the abdomen, recognizable to the position tracking system.

Following CT scanning, the radiologist determined the location of each radiological marker in the CT coordinate system. Since each marker has a three-dimensional position, the marker positions can be written in a rectangular *n* × three two-dimensional matrix, where *n* is the number of markers.

After the CT scan, each patient was asked to breathe in and out several times. Accompanying breathing movements were subject to the acquisition process using an application supported by the tracking system [27,28]. Each position measurement was marked with a time stamp.

The respiration phase is based on the analysis of the displacement of markers on the Z axis of the tracking system coordinates $\emptyset_{j}$ for any time *t_j_* has been linearly interpolated [9]:

(6) $$\emptyset_{j}=\left\{\begin{array}{c} \left(\frac{t_{j}-t_{i}^{min}}{t_{i}^{max}-t_{i}^{min}}\right)\bar{\emptyset^{max}}\;;\;for\;everyone\;t_{i}^{min}\;\leq \;t_{j}<t_{i}^{max}\\ \bar{\emptyset^{max}}+\left(\frac{t_{j}-t_{i}^{max}}{t_{i+1}^{min}-t_{i}^{max}}\right)\left(1-\bar{\emptyset^{max}}\;\right)\;;\;for\;everyone\;t_{i}^{max}\;\leq \;t_{j}<t_{i+1}^{min} \end{array},\right.$$

(7) $$\bar{\emptyset^{max}}=\frac{1}{N^{max}}\overset{N^{max}}{\underset{i=1}{\sum }}\frac{t_{i}^{max}-t_{i}^{min}}{t_{i+1}^{min}-t_{i}^{min}}$$

where: $t_{i}^{min},\;t_{i}^{max}$ indicates time corresponding to the *i*-th minimum and maximum marker position in the Z axis, respectively, and $N^{max}$ indicates the number of observed respiratory cycles.

Based on current literature, the average respiratory cycle is approximately four seconds. The maximum sampling frequency of the position tracker is 25 Hz [29]. Therefore, assuming the use of modern and efficient hardware architecture, it is possible to collect 100 sets of marker positions in one respiratory cycle.

Each patient’s metadata were saved in their patient record, comprising a summary of marker positions over time in the tracking system and a collection of marker positions in the CT coordinate system.

#### 2.1.7. Adaptation of the AQUIRC Approach to the Problem of Reducing the Time–Space Error of the Target Point during Minimally Invasive Abdominal Procedures

Datteri et al. proposed the assessing quality using image registration circuits (AQUIRC) method, which measures the quality of registration correlated with TRE [30]. The method is based on the idea of registration presented by Woods [31] and Holden [32]. Fitzpatrick proved that using only one registration chain can lead to underestimating registration errors [33]. The AQUIRC algorithm uses multiple strings to modify the marker positions with FLE estimation and then registers between the modified marker positions. Spinczyk et al. [34] achieved a significantly lower median TRE based on deformation made using spline curves and a set of successive marker positions on the surface. They assessed the correlation between FRE and TRE and compared the TRE obtained with different registration methods. The best results were obtained using the particle swarming algorithm (PSO) to calculate the best parameters for EBS spline curves.

The AQUIRC method is based on the concept of recording circuits. The registration circuit includes three basic configurations A, B, and C, and three transformations between them. In their approach, the marker positions differ between configurations by the assumed location error. In the proposed approach, the configuration coordinates vary depending on the position of the markers obtained in subsequent respiratory cycles.

A graph is created for each respiratory phase. Each vertex represents a set of marker positions obtained in the respiratory cycle. For example, *N* − 1 vertices correspond to the *N* − 1 observed respiratory cycles. The highlighted apex represents the set of markers observed on the CT scan performed before the procedure. By setting the configuration of markers in the vertices of the graph, $\left(\begin{array}{c} N\\ 2 \end{array}\right)$ edges and $\left(\begin{array}{c} N\\ 3 \end{array}\right)$ circuits are obtained.

In further analysis, those circuits with a highlighted vertex were taken into account. The registration circuit, the length of which is 3, includes three configurations, *A*, *B*, and *C*, and three transformations *T_AB_*, *T_BC_*, and *T_CA_*. The points *X* from configuration A are converted to *X*’ by using the transformation:

(8) $$X^{\prime }=T_{AB}\left(T_{BC}\left(T_{CA}\left(X\right)\right)\right)$$

where: *T_AB_*, *T_BC_*, and *T_CA_* denote transformations between configurations *A*, *B*, and *C*, respectively. Transformation between each configuration was determined by applying the rigid registration, proposed in Horn’s algorithm, depending on the marker positions [13].

Each transformation or edge of the graph is represented by the dissimilarity measure ε_A_, ε_B_, ε_C_. The difference is affected by the registration error in three configurations, i.e., the registration error between *A* and *B*, the registration error between *B* and *C*, and the registration error between *C* and *A*. With only one circuit, the contribution of each component cannot be calculated. However, it can be estimated with more than one circuit. For this purpose, it was assumed that each registration affects the quality of the multiplicative measure, i.e.,

(9) $$TRE_{obwód\_ABC}=dissimilarity\;\left(X,\;X^{\prime }\right)=\varepsilon_{A}\;\cdot \;\varepsilon_{B}\;\cdot \;\varepsilon_{c}$$

This expression can be computed for all possible circuits and transformed into a matrix:

(10) $$\left[\begin{array}{c} 1\;1\;1\;0\ldots 0\\ 1\;0\;1\;1\ldots 0\\ 1\;1\;0\;1\ldots 0\\ 0\;1\;1\;1\ldots 0\\ .\\ .\\ . \end{array}\right]\left[\begin{array}{c} \log\left(\varepsilon_{circuit_{1}}\right)\\ \log\left(\varepsilon_{circuit_{2}}\right)\\ \log\left(\varepsilon_{circuit_{3}}\right)\\ \log\left(\varepsilon_{circuit_{4}}\right)\\ \vdots \\ \log(\varepsilon_{circuit_{\left(\begin{array}{c} N\\ 2 \end{array}\right)}}) \end{array}\right]=\left[\begin{array}{c} \log\left(TRE_{circuit_{1}}\right)\\ \log\left(TRE_{circuit_{2}}\right)\\ \log\left(TRE_{circuit_{3}}\right)\\ \log\left(TRE_{circuit_{4}}\right)\\ \vdots \\ \log(TRE_{circuit_{\left(\begin{array}{c} N\\ 3 \end{array}\right)}}) \end{array}\right]$$

After solving the above system of equations, error vectors: *FRE*, *TRE*, and epsilon are calculated, in which the individual coordinates correspond to specific circuits.

#### 2.1.8. Conducted Experiments

As mentioned above, only the graph vertices that contained CT marker positions were included in further analysis. For each marker treated as a target, 50 respiratory phases (from 0 to 98% of the respiratory cycle with a 2% step) were selected for each respiratory cycle, a correspondence graph was built, and the equation system was solved. Descriptive statistics of TRE measurements were calculated, and the respiratory phase was selected based on the lowest median TRE value.

The experimental verification of the proposed methodology was carried out based on 21 patients diagnosed with liver tumors. At the stage of imaging diagnostics, a three-dimensional CT with contrast was used, depending on the adopted acquisition protocol and the potential therapeutic intervention. For example, where surgical techniques were to be used, 3D imaging was most commonly used, and the respiratory phase must be adjusted. For each patient, each marker was treated as a target and the remaining markers as target position predictors, resulting in 162 configurations and 1095 analyzed breathing cycles.

### 2.2. Synchronization of Information from Pre-Operative and Intraoperative Image Sequences

#### 2.2.1. Image Acquisition

The patient examination was conducted as follows. First, the patient was positioned supine on the CT table, and their position was aligned according to the tracker coordinate system. As reference points during the examination, radiological markers were attached to the thorax and the lateral abdominal surface. The CT table was then moved into the scanner gantry, CT examination was performed, and the table was moved back to the starting position. Four-dimensional CT and US examinations were performed in the same session, with the patient being asked to remain as still as possible during and between the examinations to minimize any motion or deformation.

CT examination was conducted on patients undergoing radiotherapy treatment with a planned 4D CT injected image (Philips Brilliance). The 4D CT reconstructed an averaged respiratory cycle from a continuous acquisition covering 10 to 15 cycles, using a helical acquisition and phase-tagging of the respiratory signal acquired via a respiratory belt.

To study the feasibility of US guidance according to the pre-operative planning CT image, US sequences were acquired in 10 patients. Ten 3D volumes were reconstructed, representing the patient’s anatomy at evenly distributed phases of respiration. Following CT scanning, several 2D US sequences of approximately 30 s, targeting structures in the liver, were obtained by the radiologist. A hand-held US probe (Clarity, Elekta) was used, which provided 2D image sequences in the coordinate system of the CT scanner using a calibrated optical tracking system. Spatial correspondence was obtained using the calibrated optical tracking system and was validated by the radiologist to obtain reasonable final results.

Two kinds of US examination were performed: a 3D sweep to reconstruct the volume of the US image, and examination at the held position of the US probe—to track breathing motion.

#### 2.2.2. Respiratory Phase Signal Estimation from US Images

After rigid registration, the CT and US data were input into the common coordinate system. After rigid registration, it was difficult to see spatial correspondence in CT and US data. The next step in analysis was to estimate the breathing signal using a free hand tracked 2D US sequence. The first step was to determine the region of interest for the whole US sequence. This was determined using three criteria. First, exclusion of pixels below the selected threshold; (USF images: 640 by 480 by 1, data type unsigned short: 0–255; default value outside the cone = 0) default: 15. Secondly, exclusion of the pixels that varied less than a specific threshold (i.e., if the corresponding pixel did not change in value in the US sequence it does not contain information about movement); default: 2. Finally, exclusion of the upper part of the US image due to potential deformation; default 120. STATISMO library was then used to transform image data into vector representation [35]. Every masked US image was one datum sample. The main components of principal component analysis and scores of main components (usually first) were calculated for every US image. The time sequence of scores estimates the breathing signal.

The next step was to estimate the breathing phase from the breathing signal using the angle of Hilbert. To validate this, the corresponding CT plane was extracted according to the detected phase and the slice orientation given by an optical device locating the probe position and orientation according to the patient. As preliminary validation, we compared the similarity between the extracted CT planes with CT planes at other time phases. Our assumption is that similarity values should be maximal for the corresponding retrieved phase and lower for the other phases, if the breathing signal extraction and the phase retrieval are correct.

#### 2.2.3. Determining the Spatio-Temporal Correspondence between the Intraoperative US Sequence and Pre-Operative the 4D CT Images

To determine the correlation between pre-operative and intraoperative data, the following method was proposed [36,37]:

- Two-dimensional CT sequences were generated from the four-dimensional CT that corresponded spatially to the US sequence;
- The breathing signal from the 2D CT sequence was estimated (based on the same method as described for US images);
- Hilbert’s transformation angles were calculated to estimate the breathing signal from the corresponding CT sequence, and the phase values were normalized in scope (0–100%);
- For every US frame, the corresponding 4D CT phase was determined by comparing the phase values.

#### 2.2.4. Validation Method

To validate the results, the CT and US images were manually segmented as follows: aorta, vena portae hepatis, vena cava inferior, and hepatic arteries. After segmentation, the results were projected to every 10 phases of 4D CT using the inverse deformation field.

## 3. Results

### 3.1. Modeling of Respiratory Movements as a Problem of Time–Space Adjustment

The respiratory phase was selected using the approach described above. In 17 out of the 21 patients, the optimal phase selected in the AQUIRC experiment coincided with the respiratory phase according to the markers lying in the center of the system.

**Figure 2:**
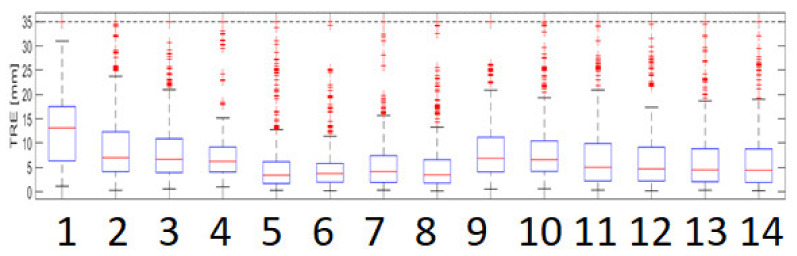
Summary of the distribution in the AQUIRC respiratory group with division into the applied transformation types and optimization methods (legend: TRE_RIGID_ 1, TRE_AFFINE_ 2, TRE_RIGID-TPS_ 3, TRE_AFFINE-EBS_ 4, TRE_RIGID-EBSPSO_ 5, TRE_RIGID-EBSPSO-AQUIRC_ 6, TRE_RIGID-EBSDE_ 7, TRE_RIGID-EBSDE-AQUIRC_ 8, TRE_AFFINE-TPS_, TRE_AFFINE-EBS_ 9, TRE_AFFINE-EBS_ 10, TRE_AFFINE-EBSPSO_ 11, TRE_AFFINE-EBSPSO-AQUIRC_ 12, TRE_AFFINE-EBSDE_ 13, and TRE_AFFINE-EBSDE-AQUIRC_ 14).

The summary of the TRE for the respiratory phases found using the AQUIRIC method is as follows (see Figure 2). The minimum TRE was obtained for TRE_RIGID-EBSDE-AQUIRC_ = 3.47 and TRE_RIGID-EBSPSO-AQUIRC_ = 3.73 mm, and intermediate values TRE_AFFINE-EBSDE-AQUIRC_ = 4.41 and TRE_AFFINE-EBSPSO-AQUIRC_ = 4.7 and the greatest TRE_AFFINE_ = 7.0 and TRE_RIGID_ = 13.13. Figure 1 is a box plot showing an overview of the TRE values obtained.

### 3.2. Synchronization of Information from Pre-Operative and Intraoperative Images

**Figure 3:**
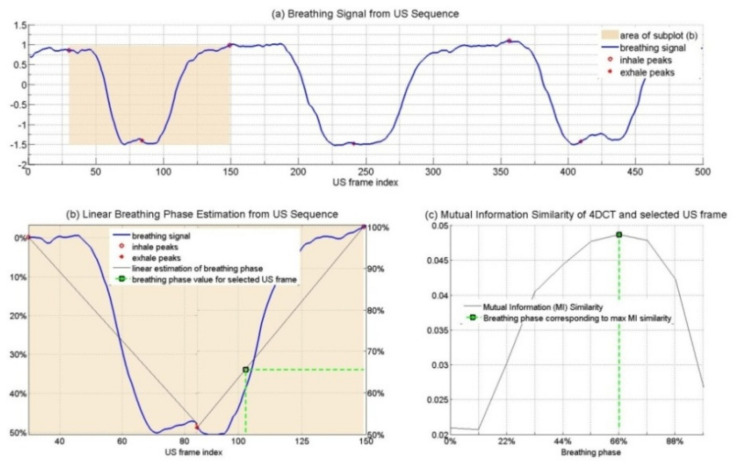
Extracted breathing signal as a function of US frames (**a**). Linear breathing phase estimation from US sequence (**b**). Mutual information as a function of CT phases (**c**).

Figure 3 depicts the similarity measure (mutual information) [38] between 10 planes extracted from the 4D CT corresponding to the US probe orientation at a given frame. We can observe that the maximum value corresponds to the one extracted by the proposed method.

**Figure 4:**
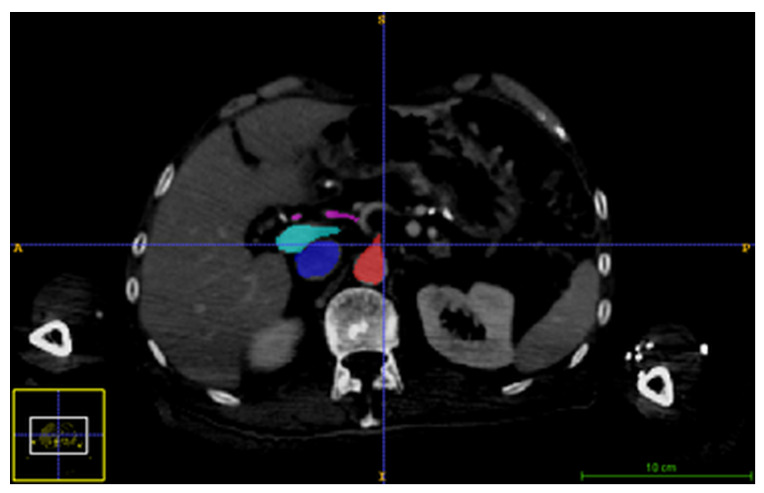
An example of segmented inferior vena cava (dark blue), vena portae hepatis (light blue), and aorta (red).

**Figure 5:**
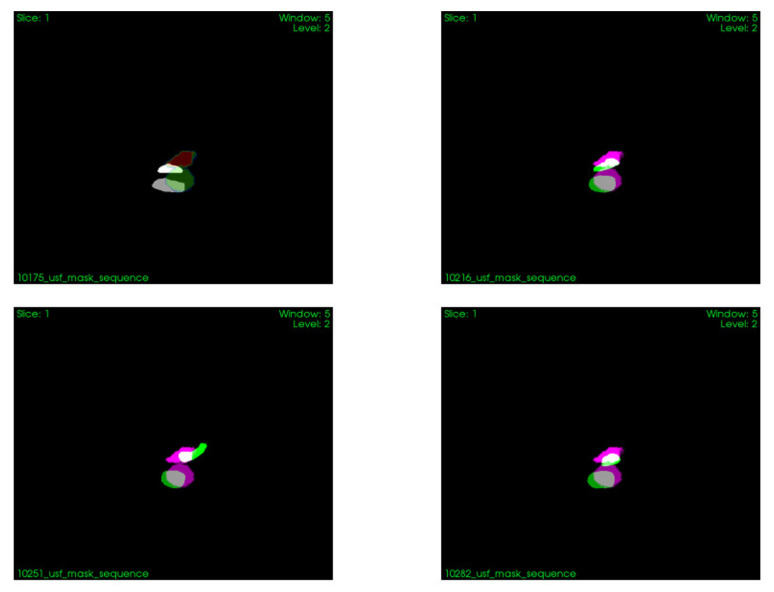
Overlay of the binary masks of the segmented vena cava and portal vein in US (white) and CT images (pink) for: exhale (**top-left**), deflation (**top-right**), inhale (**bottom-left**), and inflation (**bottom-right**).

An example of segmentation results in a CT image is shown in Figure 4 and binary image fusion in Figure 5. Images are presented without manual spatial correspondence alignment. The average maximum distance was 3.4 (minimum: 1.6, maximum 6.8) mm.

## 4. Discussion

When analyzing the obtained results in modeling spatio-temporal deformation, the smallest absolute error value when estimating the position of the tracked lesion was obtained using the RIGID-EBSPSO method. However, the approach based on observing the marker error in the respiratory cycle is difficult to apply in clinical conditions. Furthermore, in the current research, the position of the minimum respiratory error occurred in various respiratory phases [33]. Therefore, considering future clinical applications, this study focused on methods to determine the respiratory phase based on recording circuits. This method allows the determination of a constant respiratory phase for which the lowest error in the estimation of the location of the pathological change was observed.

The analysis was performed on free-breathing data collected in preparation for minimally invasive abdominal surgery. In real clinical scenarios, such interventions are performed under general anesthesia, and the breathing process is supervised by a ventilator, resulting in regular and predictable breathing movements. However, there are some limitations to this approach. First, to confirm the true target position and to minimize invasiveness, matched abdominal surface markers were used as targets. In the future, the proposed approach may include a marker placed inside the abdominal cavity. Second, experiments with animal tissues during the respiratory cycle showed that the relative position of the markers and the direction of their distribution influenced the accuracy of the TRE assessment [39].

Referring to the limitations of the proposed method and the conducted experiments, it should be emphasized that the method is ultimately planned to use the estimation of the location of the pathological lesion in the liver. Due to the limitations of the consent of the Ethics Committee regarding the invasiveness of the conducted experiments and the need to ensure accurate knowledge of the location of the target point, the scope of the target point location was limited in the research to a marker tracked by the position tracking system located on the surface of the patient’s abdominal cavity. In the future, it is planned to extend the experiments to patients who use internal markers in the liver that unambiguously locate a pathological change inside the organ.

In minimally invasive surgery, one of the main challenges is precisely locating the target during surgical intervention [40]. In the case of abdominal intervention, one of the most important factors affecting the target’s movement is breathing. The aim of the study was to efficiently forecast the target’s location in the respiratory cycle. The lowest median TRE error in the recording circuits was observed for markers located in the center of the coordinate system, which is consistent with the Fitzpatrick estimator theory [41]. The obtained set of CT images was not subject to strict control of the acquisition protocol in terms of respiratory phases; therefore, we do not know whether individual examinations of patients were carried out during inhalation or exhalation, hence the phases determined by the method may be from different points in the respiratory cycle. If it were possible to perform the research from scratch, it would be prudent to establish an imaging protocol that would consider the reproducibility of CT images. From a clinical point of view, exhalation is easier to control and more reproducible. High-frequency jet ventilation can also be used to minimize respiratory movements, though this is limited by the availability of the apparatus and general condition of the patients health [42].

As demonstrated in the studies above, it is possible to estimate the respiratory phase directly from the sequence of US images and use this signal to time and synchronize intraoperative US images with the pre-operative 4D CT sequences that model the respiratory movements of the liver organ. By selecting the region of interest in the US image around the liver tumor to be treated, a local respiratory phase signal based on internal organ displacement can be obtained. Synchronization of this information is important when the neoplastic lesion is poorly or not visible on the intraoperative US images and, therefore, there is an objective difficulty in locating the neoplastic lesion [4,5].

## 5. Conclusions

The real-time method using skin markers and non-rigid registration algorithms has been implemented and tested for various classes of transformation, which purpose was estimating the displacement of the target point. The average computation time for this method is less than 200 ms. In addition, the possibility of estimating the respiratory phase signal directly from intraoperative US images and the possibility of synchronization with the 4D CT respiratory sequence are also presented, based on ten patients.

The article presents an analysis of the two methods proposed above independently. However, when viewed from a clinical point of view, a combination of the two may be useful. Pre-rigid registration of the pre-operative CT volume and the patient’s position during the procedure allows for a coarse localization of the lesion. The method of synchronizing the pre-operative and intraoperative images allows the determination of the initial position of the US image on which the change should be visible. Then, the method of modeling spatio-temporal deformations can be used to estimate the region of interest, to narrow down the location of the neoplastic lesion on the US images. The described approach will form the basis for further research, which will allow for a quantitative evaluation of the proposed combination of the two presented methods.
